# A novel bacterial strain *Burkholderia* sp. F25 capable of degrading diffusible signal factor signal shows strong biocontrol potential

**DOI:** 10.3389/fpls.2022.1071693

**Published:** 2022-11-24

**Authors:** Hongxiao Yu, Wen-Juan Chen, Kalpana Bhatt, Zhe Zhou, Xixian Zhu, Siqi Liu, Jiehua He, Lian-Hui Zhang, Shaohua Chen, Huishan Wang, Lisheng Liao

**Affiliations:** ^1^ Department of Plant Pathology, College of Plant Protection, South China Agricultural University, Guangzhou, China; ^2^ Guangdong Province Key Laboratory of Microbial Signals and Disease Control, Integrative Microbiology Research Centre, South China Agricultural University, Guangzhou, China; ^3^ Department of Botany and Microbiology, Gurukula Kangri University, Haridwar, Uttarakhand, India

**Keywords:** diffusible signal factor (DSF), quorum quenching, quorum sensing, Burkholderia, biocontrol, plant diseases

## Abstract

Vast quantities of synthetic pesticides have been widely applied in various fields to kill plant pathogens, resulting in increased pathogen resistance and decreased effectiveness of such chemicals. In addition, the increased presence of pesticide residues affects living organisms and the environment largely on a global scale. To mitigate the impact of crop diseases more sustainably on plant health and productivity, there is a need for more safe and more eco-friendly strategies as compared to chemical prevention. Quorum sensing (QS) is an intercellular communication mechanism in a bacterial population, through which bacteria adjust their population density and behavior upon sensing the levels of signaling molecules in the environment. As an alternative, quorum quenching (QQ) is a promising new strategy for disease control, which interferes with QS by blocking intercellular communication between pathogenic bacteria to suppress the expression of disease-causing genes. Black rot caused by *Xanthomonas campestris* pv. *campestris* (*Xcc*) is associated with the diffusible signal factor (DSF). As detailed in this study, a new QQ strain F25, identified as *Burkholderia* sp., displayed a superior ability to completely degrade 2 mM of DSF within 72 h. The main intermediate product in the biodegradation of DSF was identified as n-decanoic acid, based on gas chromatography-mass spectrometry (GC-MS). A metabolic pathway for DSF by strain F25 is proposed, based on the chemical structure of DSF and its intermediates, demonstrating the possible degradation of DSF *via* oxidation-reduction. The application of strain F25 and its crude enzyme as biocontrol agents significantly attenuated black rot caused by *Xcc*, and inhibited tissue maceration in the host plant *Raphanus sativus* L., without affecting the host plant. This suggests that agents produced from strain F25 and its crude enzyme have promising applications in controlling infectious diseases caused by DSF-dependent bacterial pathogens. These findings are expected to provide a new therapeutic strategy for controlling QS-mediated plant diseases.

## Introduction

Quorum sensing (QS) is commonly observed in microorganisms regulating biofilm formation and cell growth (Hammond et al., 2015; Watve et al., 2020; Mishra et al., 2022). It also facilitates important biological functions such as extracellular product synthesis, bioluminescence, and the production of virulence factors (Semighini et al., 2006; Deng et al., 2014; Bukvicki et al., 2016). Population sensing is a communication mechanism between bacteria, referring to the process by which bacteria adjust their population density and behavior by sensing the levels of various signaling molecules in their environment (Ya'ar Bar et al., 2021; Liu et al., 2022; Zhou et al., 2022). Changes in the species composition and cell density of microbial communities can transmit information through complex signaling systems, allowing the bacteria to collectively change their behavior by exchanging information among themselves (Wang et al., 2004; Whiteley et al., 2017). The diffusible signal factor (DSF) family QS system is a conserved cellular communication system widely found in Gram-negative bacteria, involved in regulating the production of toxic substances by Gram-negative bacteria (Deng et al., 2016; Cui et al., 2018; Ye et al., 2019). The DSF family QS system has been confirmed to exist in a variety of *Xanthomonas* species (Bi et al., 2014; Kanugala et al., 2019), such as *Xanthomonas campestris* pv. *campestris* (*Xcc*) (Ryan et al., 2015), *Xanthomonas oryzae* pv. *oryzae* (*Xoo*) (Chatterjee and Sonti, 2002), *Xanthomonas axonopodis* pv. *citri* (*Xac*) (Deng et al., 2011), and *Xanthomonas axonopodis* pv. *glycines* (*Xag*) (Park et al., 2019). Among them, *Xcc* causes cruciferous black rot, a plant disease that has a significant impact worldwide and has consequently attracted a lot of attention (Zhou et al., 2017; Rubel et al., 2019).

Black rot caused by *Xcc* occurs in vegetables in the cruciferous family such as radishes, cabbage, mustard, cauliflower, and kale (Mishra and Arora, 2012). The black rot pathogen usually multiplies and spreads rapidly in the field in warm and humid climates, causing black rot on the young stems and leaves of the plants, resulting in a severe reduction in crop quality and yield (An et al., 2020). In actual production, the control measures taken against the causative agent of black rot mainly include chemical control, such as chlorothalonil, mancozeb, agricultural streptomycin, among others (Molitor and Beyer, 2014; Sreelatha et al., 2022). It is well-known that the massive use of chemical pesticides causes serious environmental pollution, a series of safety problems (e.g., food safety), and affects human health (McEwen and Collignon, 2018; Ye et al., 2020c). Indiscriminate antibiotic use may lead to more pathogenic bacteria developing specific or even multi-drug resistance, hindering the control of diseases such as black rot (Agerso et al., 2007; Alsan et al., 2015; George, 2018). Therefore, it is urgent to develop environmentally friendly and efficient control strategies (Majumdar and Pal, 2016; Mukherjee and Bassler, 2019).

A series of extracellular enzymes produced by *Xcc* play an important role in the pathogenesis of the bacteria after the host plant is infected (Hsiao et al., 2010; Timilsina et al., 2020; Islam et al., 2021). The signaling molecule DSF has been identified as *cis*-11-methyl-2-dodecenoic acid and was associated with the pathogenic process caused by *Xcc* (He and Zhang, 2008; Deng et al., 2015; Zhou et al., 2015). Suppressing the expression of pathogenic genes during QS through blocking intercellular communication between pathogenic bacteria interferes with QS and has been recognized as a highly promising disease control measure, also known as quorum quenching (QQ) (Gu et al., 2018; Xu et al., 2021; Wang et al., 2022). QQ is a new strategy for disease control, proposed based on QS. It acts as a biological control by inhibiting the synthesis, accumulation, and monitoring of signaling molecules (Nhan et al., 2010). The quorum sensing system can also be interfered with through enzymatic degradation or modification of the signaling molecules (Zhang et al., 2019). In this way, the goal of inhibiting the expression of genes related to the pathogenicity of microorganisms can be achieved, thus attenuating their pathogenicity (Zhang, 2003). Ultimately, the purpose of disease control can be achieved. Degradation of microbial signaling molecules using quenching sterilization or quenching enzymes is, at present, the least toxic and most effective pathway for quorum quenching (Chen et al., 2013; Bhatt et al., 2022). QQ is carried out by regulating the QS system to control diseases and does not produce selection pressure on microorganisms, such that the pathogenic bacteria do not develop resistance (Steindler and Venturi, 2007). There are currently three methodological approaches to QQ: the first is based on quorum sensing inhibitors (QSIs), such as the inhibitor halogenated furanones first identified from marine red algae (*Deliseapulchra*) (Wopperer et al., 2006), whose mechanism is to inhibit the synthesis of the signaling molecule (Kim et al., 2007); the second involves the use of a structural analogue of the signaling molecule, whose mechanism is to interfere with the binding of the signaling molecule to the receptor protein by competitively binding to the corresponding receptor protein (Murugayah and Gerth, 2019); and the third is a quenching molecule or quenching enzyme, whose mechanism is to degrade the signaling molecule such that it does not reach a certain threshold value (Hong et al., 2012; Rolland et al., 2016). Overall, quorum quenching or quenching enzymes acting outside the cell can avoid (or, at least, reduce) the selection pressure on cells, compared to inhibitors of signaling molecules. With more in-depth studies, QQ pathways based on quenching and sterilizing or quenching enzymes for plant disease control are expected to achieve significant breakthroughs that cannot be achieved by traditional chemical control means (Turan et al., 2017; Leguina et al., 2018; Wang et al., 2020).

Many Gram-negative bacteria rely on the QS system to detect their population density and activate the expression of some relevant genes by releasing an accumulation of signaling molecules (Zhang et al., 2020). There are two main approaches to quorum quenching sterilization with biocontrol effects. On one hand, quorum quenching enzyme genes can be transferred into microorganisms to obtain transgenic quenching sterilization (Dong and Zhang, 2005). On the other hand, quorum quenchers can be screened from nature, as many microbial taxa can degrade *N*-acyl homoserine lactone (AHL) signaling molecules (Huang et al., 2016; Wang et al., 2022). Recently, several microbial strains such as *Acinetobacter lactucae* QL-1 (Ye et al., 2019), *Pseudomonas* sp. HS-18 (Wang et al., 2020), and *Cupriavidus pinatubonensis* HN-2 (Xu et al., 2021) capable of degrading DSF have been isolated and characterized. However, genetically engineered quenchers have not yet been widely accepted, as they are not easy to cultivate. In contrast, there exist a large number of microorganisms in nature that can degrade signaling molecules, which are easy to cultivate in large numbers (Ye et al., 2020a; Ye et al., 2020b). Therefore, screening populations for quenching sterilization from nature present more obvious advantages for application as a biodegradation agent.

In this study, a strain of *Burkholderia* sp. F25 is identified as a significant degrader of DSF, and the degradation mechanism, degradation products, and degradation capacity of F25 are investigated. This bacterium can degrade DSF rapidly and efficiently, presents a quorum quenching function, and has a significant biological effect on the prevention of DSF-dependent diseases, such as black rot caused by *Xcc* XC1. In addition, the properties and biocontrol effects of crude enzymes extracted from F25 are investigated. This study, considering strain F25 as a biocontrol agent, provides new ideas to clarify the pathogenic regulatory mechanisms of DSF signaling-mediated pathogens, as well as new insights for the development of biocontrol strategies against DSF signaling-mediated bacterial pathogens.

## Materials and methods

### Chemicals and plants

Diffusible signal factor (DSF) (≥99%) was purchased from Shanghai UDChem Technology Co., Ltd (Shanghai, China) and dissolved in methanol to create a stock solution with a concentration of 100 mmol·L^-1^. Radishes (*Raphanus sativus* L.) were purchased from a local market (Guangzhou, China) and healthy plants were selected for the biocontrol experiments.

Ampicillin (AMP, 50 mg·mL^-1^), gentamicin (GEN, 50 mg·mL^-1^), neomycin sulfate (NEO, 50 mg·mL^-1^), carbenicillin (CARB, 50 mg·mL^-1^), chloramphenicol (CM50, 30 mg·mL^-1^), tetracycline (TC, 5 mg·mL^-1^), kanamycin (KAN, 50 mg·mL^-1^), and streptomycin (STR, 50 mg·mL^-1)^, were used for antibiotic susceptibility testing, purchased from Sigma Aldrich Chemicals Co., Ltd (Shanghai, China).

### Strains and culture conditions

XC1 was provided by the Integrative Microbiology Research Centre, South China Agricultural University, Guangzhou, China. *Xcc* were cultured on Luria–Bertani (LB) medium (NaCl 10.0 g·L^-1^, tryptone 10.0 g·L^-1^, and yeast extract 5.0 g·L^-1^) with rifampicin (30 μg·mL^-1^) at 28°C. The isolates were grown on LB medium or mineral salt medium (MSM: (NH_4_)_2_SO_4_ 2.0 g·L^-1^, Na_2_HPO_4_·12H_2_O 1.5 g·L^-1^, KH_2_PO_4_ 1.5 g·L^-1^, MgSO_4_·7H_2_O 0.2 g·L^-1^, CaCl_2_·2H_2_O 0.01 g·L^-1^, FeSO_4_·7H_2_O 0.001 g·L^-1^; pH 7.2) with DSF (2 mmol·L^-1^) at 30°C. The MSM medium and minimal medium (MM: (NH_4_)_2_SO_4_, 2.0 g; MgSO_4_·7H_2_O, 0.2 g; CaCl_2_, 0.01 g; FeSO_4_, 0.005 g; MnCl_2_, 0.002 g; K_2_HPO_4_, 10.5 g; KH_2_PO_4_, 4.5 g; mannitol, 2.0 g; glycerol, 2.0 g; 1000 mL of H_2_O; pH 6.5) were used to test the degradation efficiency of strain F25 on diffusible signal factor (DSF) and for the identification of metabolites.

### Isolation and screening of *Burkholderia* sp. strain F25

Soil samples were collected on March 16, 2017, from the surface layer to a depth of 5 cm, at a perennially cultivated sweet potato field in Heshun Lugang, Nanhai District, Foshan City, Guangdong Province (Longitude: 113.14299°; Latitude: 23.02877°). The soil was sampled, bagged, and preserved as a microbial source for strain isolation. MSM medium was prepared, and 50 mL of MSM medium was sterilized in 250 mL triangular flasks. After cooling, DSF mother liquor (mother liquor concentration, 100 mM; methanol as solvent) was added under aseptic conditions, to make the final mass concentration of DSF 0.01 mM. Then, 5 g of soil sample was added, and the solution was incubated at 30°C in a 200 rpm shaker for 7 d. After this, it was transferred to the second batch of MSM medium, with a final mass concentration of DSF of 100 μM at 10% inoculum. After 7 d of incubation under the same conditions, the sample was transferred to MSM medium with a final mass concentration of 200 μM DSF at 10% inoculum, and incubated for another 7 d. The mass concentration of DSF was increased continuously. Then, 1 ml of MSM medium fermentation broth was diluted with sterile water into 10^-1^, 10^-2^, 10^-3^, 10^-4^, 10^-5^, and 10^-6^ fermentation broths in a concentration gradient. The mass concentration of DSF was continuously increased through this method, and the final enrichment of the strain was achieved. Then, 100 μL of diluted fermentation broth from each concentration gradient was evenly spread on LB solid plates and incubated at 30°C in an incubator, and single colonies were picked. The LB solid plate was repeatedly scribed and purified until a single strain was isolated (Bhatt et al., 2020; Zhang et al., 2022). The isolated single strains were stored in a refrigerator at −80°C.

Single colonies of the purified strains were inoculated in 40 mL of MSM basal medium with DSF as the sole carbon source, resulting in a final mass concentration of 2 mM DSF, then incubated at 30°C for 48 h in a 200 rpm shaker for DSF extraction and high-performance liquid chromatography (HPLC) determination of DSF residues. The strain with the highest DSF degradation rate was finally obtained, which was named F25.

### Identification of *Burkholderia* sp. strain F25

The morphological characteristics of the colonies were studied. Strain F25 was scribed in LB solid medium and incubated at 30°C for 48 h. The genome of strain F25 was extracted as a template and 16S rDNA PCR amplification of the strain was performed using bacterial universal primers 27F (AGAGTTTGATCCTGGCTCAG) and 1492R (GGTTACCTTGTTACGACTT) (Li et al., 2022). The 16S rDNA gene sequence of strain F25, with a length of 1407 bp, was obtained using the above method and then compared with the NCBI (National Center for Biotechnology Information) database (http://www.NCBI.nlm.nih.gov/).

### Antibiotic susceptibility analysis of strain F25

The antibiotic susceptibility of strain F25 was studied to better investigate the biocontrol potential of strain F25. A total of 50 μL of the bacterial solution was added to 5 mL of LB liquid medium, followed by the addition of antibiotics, where the final concentration gradient was 5, 10, 20, 50, 100, 150, 200, 250, 300, 350, and 400 μg·mL^-1^. Three sets of replicate trials were set up for each concentration gradient. Cultures were incubated at 30°C and 200 rpm for 16–24 h before measuring and recording the OD_600_ values of the cultures.

### Determination of the relationship curve between growth and degradation DSF of strain F25

A single colony of strain F25 was inoculated in LB medium and pre-cultured to the logarithmic phase, following which the resulting broth was centrifuged at 4000 rpm for 5 min. The supernatant was discarded, and the organism was washed in 0.9% sterile saline, then re-suspended as seed suspension. The bacteria were then inoculated into 50 mL of MSM basal medium at an inoculum of 1:100 and DSF was added to a final concentration of 2 mM. The culture was incubated at 30°C for 72 h at 200 rpm, and samples were taken at regular intervals. The samples were collected at different time points, and the OD_600_ value was measured spectrophotometrically to indicate the growth of strain F25, while the residual amount of DSF was measured by HPLC to indicate the degradation of DSF by strain F25.

### Antagonistic test of F25 and pathogenic bacteria

To study the antagonistic effect of strain F25 with pathogenic bacteria, studies were carried out on LB solid plates (Li et al., 2020; Zhou et al., 2022). A bacterial solution of *Xcc* was inoculated at 10% into the melted LB. After cooling, the LB agar plates containing the pathogen were punched with an inactivated punch, and 20 μL each of bacterial solution, metabolite solution, acetonitrile, and sterile water of strain F25 were injected into the punched wells. The plates were incubated at 30°C for 24 h. If antagonism occurred, hyaline circles would appear in the LB plate.

### Study on the effect of strain F25 on the biological control of black rot of radishes

Single colonies of strain F25 and XC1, a DSF-dependent pathogen, were isolated and pre-cultured in LB medium until the logarithmic phase. The resulting broth was centrifuged at 4000 rpm for 5 min, and the supernatant was discarded. The organisms were washed with 0.9% sterile saline and re-suspended as seed suspension. The bacteria were then inoculated into LB medium at an inoculum of 1:100 and incubated at 30°C and 200 rpm until the logarithmic phase. The bacteria were re-suspended in PBS buffer to obtain suspensions of strain F25 and XC1.

The bacterial suspension of strain F25 was mixed with the suspension of XC1 to obtain the mixed bacterial solution. The fleshy roots of white radish were washed with distilled water, then sliced when the surface was dry. The fleshy roots were sliced crosswise to obtain round slices about 0.3 cm thick and placed in Petri dishes (with cotton moistened with sterile water). The OD_600_ of both strain F25 and XC1 was 0.2. The mixture was coated with a spreading rod and incubated at 30°C for 48 h to observe the incidence. XC1 alone and F25 alone were used as positive and negative controls, respectively. In total, there were four experimental groups, divided into XC1+sterile water, XC1+F25+sterile water, F25+sterile water, and sterile water. In the trays in which the radish slices were placed, we included moistened sterile wet water sponges and the trays were sealed with cling-film. The experimental procedure was repeated at least three times for each group. Incubation was carried out at 28°C for 48 h. The extent of the lesion was quantified by measuring the area of maceration, compared to the tissue without the lesion before inoculation (Liao et al., 2015; Liu et al., 2017).

### Biocontrol experiments on the effect of *Burkholderia* sp. F25 crude enzyme on Xcc

The crude enzyme solution of strain F25 was prepared by the following method. The overnight culture of strain F25 was centrifuged at 4°C for 10 min at 10,000 rpm, and the resulting supernatant was the extracellular enzyme. Washing and suspending the cellular sediment was achieved by rinsing it three times with PBS. The cell suspension of strain F25 was then sonicated and centrifuged. The supernatant obtained was the intracellular enzyme to be collected. To verify its effect on the control of *Xcc* black rot, experiments were performed with the resulting crude enzyme extract, with four designs: (1) XC1-treated radish slices; (2) radish slices treated with 1 µl of XC1 and intracellular enzyme of strain F25; (3) treatment with 1 µl of extracellular enzyme of strain F25 and XC1 radish slices; and (4) radish slices treated with sterile water as a control. Three replicate trials were performed for all treatments. A final assessment of the disease level severity was performed.

### Acylase activity test

To determine whether strain F25 presents acylase activity (Bao et al., 2020), we analyzed the degradative enzymes of F25 using an Acylase Activity Assay Kit (Solarbio, Beijing Solarbio Science & Technology Co., Ltd., China). Strain F25 was incubated in LB for 12 h. Intracellular and extracellular enzymes of strain F25 were extracted and collected separately. Four experimental groups were designed, with one intracellular enzyme and one extracellular enzyme setup, a negative control group without enzyme addition, a blank control group without enzyme addition, and a blank control group with distilled water. The acylase catalyzed the transfer of the acetyl group of acetyl coenzyme A to butanol and the simultaneous reduction of 5,5’-dithiobis-(2-nitrobenzoic acid) (DTNB), to produce 2-nitro-5-thiobenzoic acid (TNB), which is a yellow compound.

### Statistical analysis

Experimental data were analyzed by one-way analysis of variance (ANOVA), and means were compared by Bonferroni’s multiple comparison test using the GraphPad Prism software (Version 6.0). Experiments were arranged as a completely randomized design, and *P* < 0.05 was considered to indicate statistical significance.

## Results

### Isolation, screening, and identification of strain F25

In this study, strains with DSF degradation ability were screened from the collected soil samples by enrichment culture. MSM with DSF as the sole carbon source was prepared and strains from different soil samples were enriched in the MSM. Nine morphologically different strains capable of degrading DSF were attained by streaking plate method and named as F20~28, respectively. Among them, strain F25 with rapid DSF degradation potential was selected and used in further studies. The F25 strain was deposited at the Guangdong Microbial Culture Collection Center (GDMCC) with the collection number GDMCC NO: 60346.

**Figure 1:**
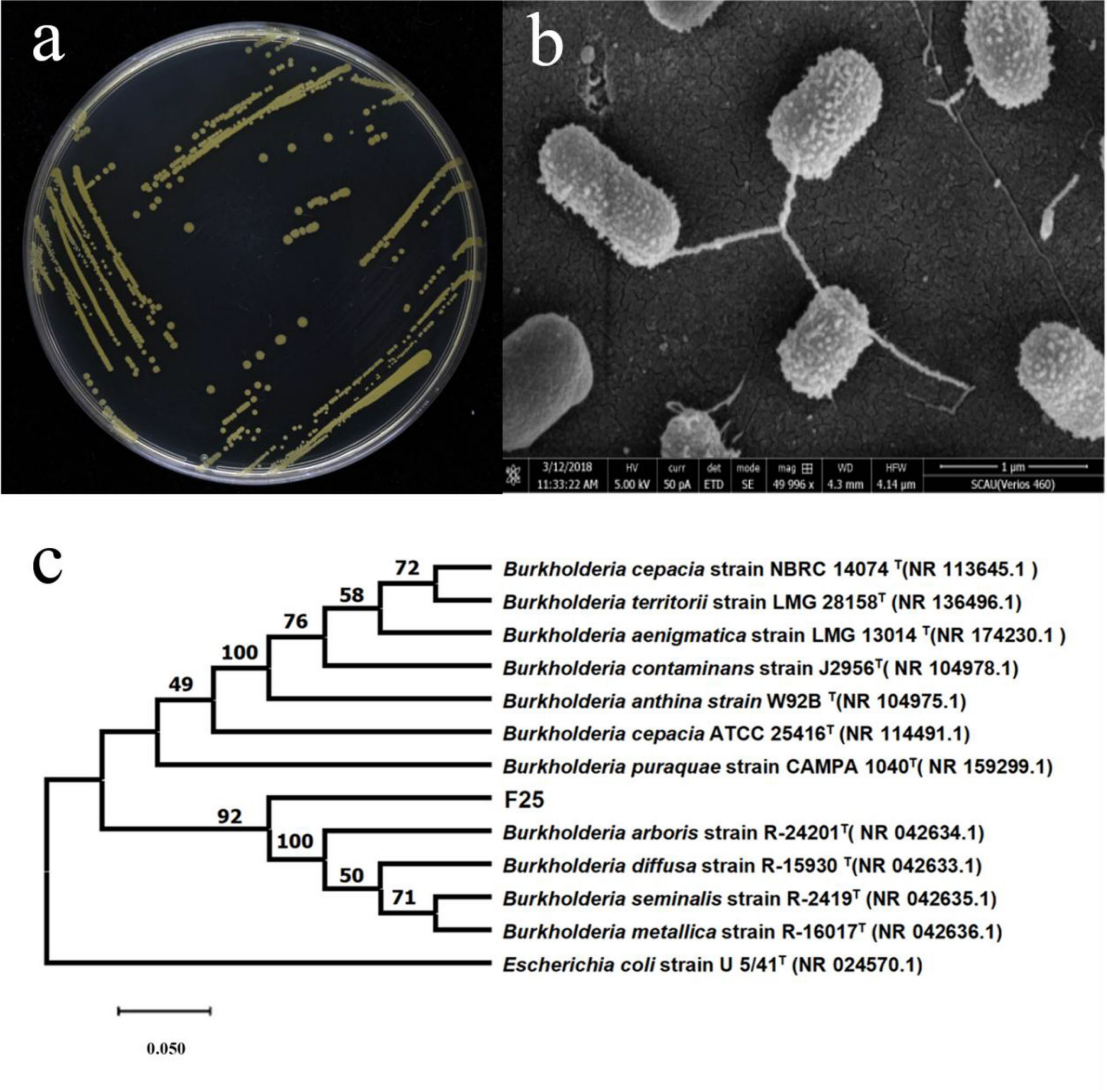
**(A)** Morphological characteristics of strain F25; **(B)** morphological characteristics observed under the scanning electron microscope (5000×); **(C)** Phylogenetic tree based on 16S rDNA sequences of *Burkholderia* sp. F25. Numbers in parentheses represent GenBank accession numbers. Numbers at nodes indicate bootstrap values. The bar represents sequence divergence.

The above strain F25 was scribed in LB solid medium and incubated at 30°C for 48 h. As shown in **Figure 1A**, the colonies were yellowish-green in color, slightly elevated, and had smooth and opaque surfaces and neat edges. Strain F25 was diffusely turbid and aerobic in LB liquid medium. Scanning electron microscopy indicated that the strain was rod-shaped (or short spherical), with a size of 0.5–1.0 × 0.3–0.5 μm (**Figure 1B**).

The genome of strain F25 was extracted as a template, and 16S rDNA PCR amplification of the strain was performed using bacterial universal primers, in order to obtain the 16S rDNA gene sequence of strain F25, which was 1407 bp in length. It was compared with the NCBI database (http://www.NCBI.nlm.nih.gov/), and we found that strain F25 had 99% high homology with *Burkholderia cepacia* TC62 (AY677087.1). The standard strains in the BLAST results were compared, and a phylogenetic tree was constructed using MEGA 6.0. The constructed phylogenetic tree is shown in **Figure 1C**.

**Figure 2:**
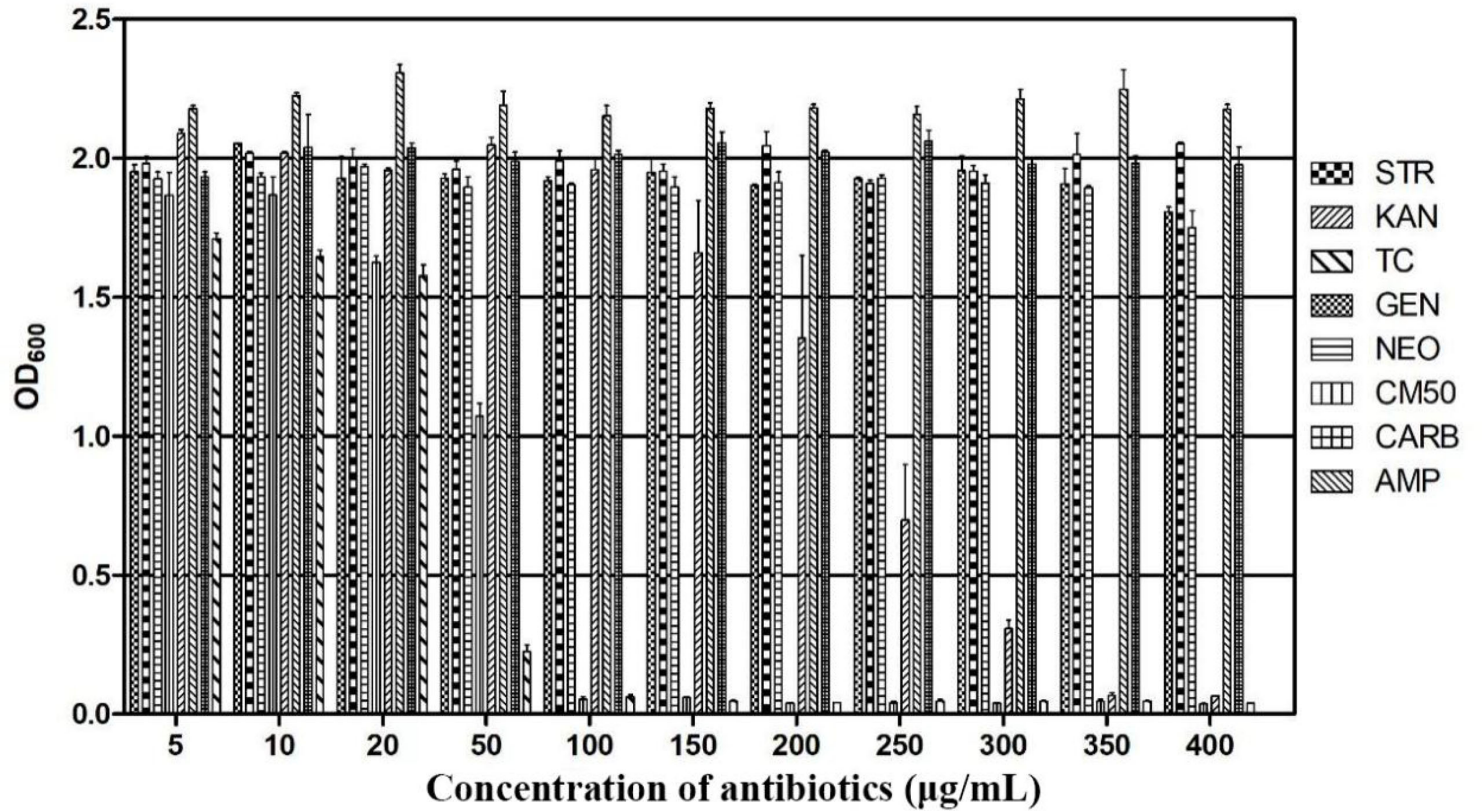
Antibiotic sensitivity of *Burkholderia* sp. F25. Strain F25 was resistant to 400 μg·mL^-1^ or more of gentamicin (GEN), neomycin (NEO), carbenicillin (CARB), ampicillin (AMP), and streptomycin (STR); 300 μg·mL^-1^ of kanamycin (KAN); and 50 μg·mL^-1^ of tetracycline (TC) and chloramphenicol (CM50).

Therefore, strain F25 was identified as *Burkholderia* sp., in accordance with its morphological characteristics, 16S rDNA gene sequence, and phylogenetic analysis. The antibiotic susceptibility of strain F25 was further investigated. The results showed that strain F25 was resistant to 400 μg·mL^-1^ or more of gentamicin, neomycin, carbenicillin, ampicillin, and streptomycin; 300 μg·mL^-1^ of kanamycin; and 50 μg·mL^-1^ of tetracycline and chloramphenicol (**Figure 2**).

### DSF degradation kinetics

**Figure 3:**
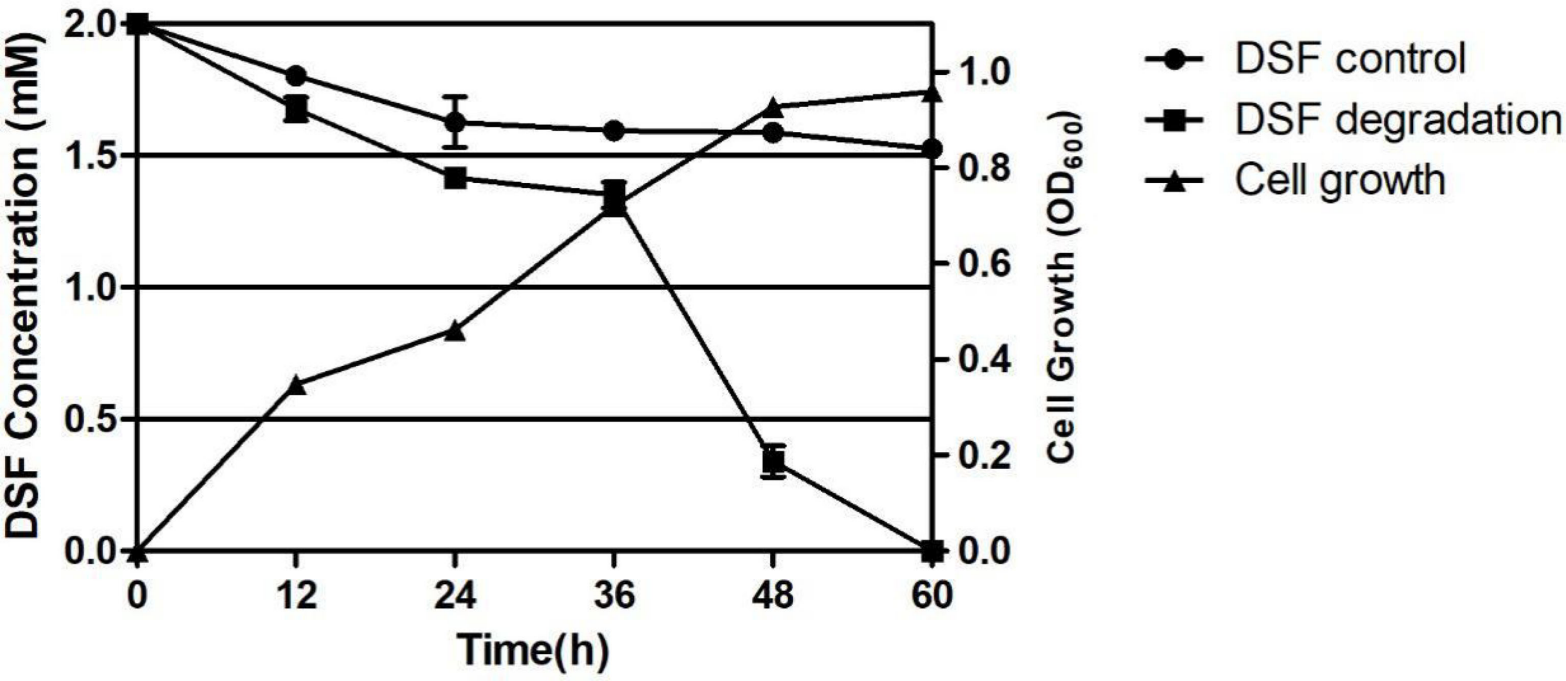
Degradation of diffusible signal factor (DSF) during the growth of *Burkholderia* sp. F25. Each experiment was conducted with three replicates. Bars indicate the standard deviation of the mean.

The DSF degradation ability of the strains was tested by growing the strains in MSM spiked with DSF. The solution was extracted at regular intervals and stored for the detection of residual DSF. The HPLC results are shown in **Figure S1**, from which it can be seen that the amount of DSF decreased with time and finally disappeared completely. At 12, 24, 36, 48, and 60 h, strain F25 degraded DSF up to 16.35%, 29.20%, 32.63%, 83.00%, and 100%, respectively. The growth and degradation curves of the corresponding strain F25 with DSF as the only carbon source are shown in **Figure 3**, where the degradation of DSF was positively correlated with the growth of the strain. In the presence of DSF, the strain growth had no lag period and rapidly entered the logarithmic phase of growth. The fastest phase of DSF degradation by this strain was 36-48 h and, when the strain was cultured to 60 h, the DSF was completely decomposed. The natural degradation rate of DSF in the control was 20% within 60 h.

### Degradation products and pathways of strain F25

**Figure 4:**
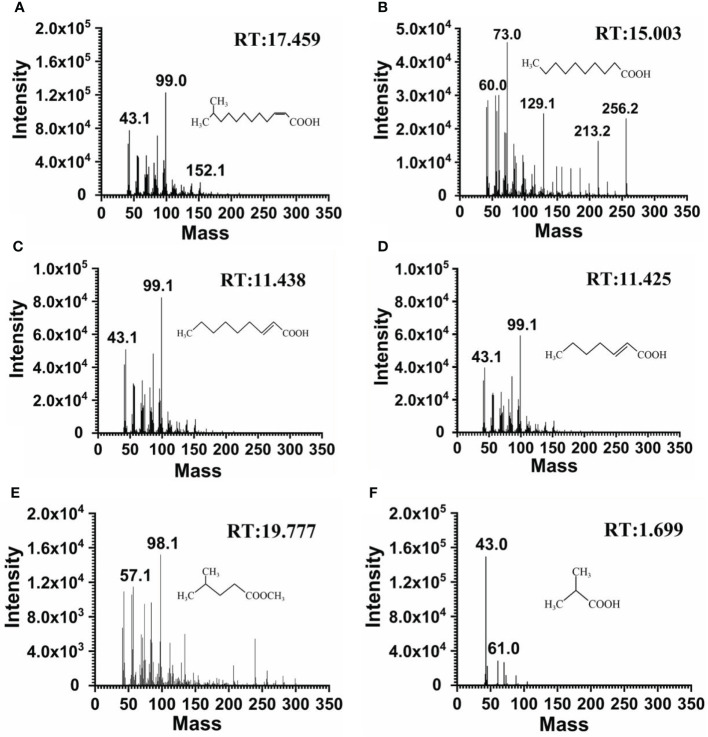
Mass spectrometric analysis of the DSF degradation products of *Burkholderia* sp. F25. GC-MS spectrum showed DSF *(m*/*z* of M+H = 99.0) with an HPLC retention time (RT) of 17.459 min **(A)**. GC-MS spectrum showed the major intermediate product *N*-decanoic acid (*m*/*z* of M+H = 73.0) with an HPLC retention time (RT) of 15.003 min **(B)**. GC-MS identified the mass spectra and structures of the other degradation products detected: *trans-*2-nonenoic acid **(C)**; *trans-*2-heptenoic acid **(D)**; methyl 4-methylpentanoate **(E)**; and 2-methylpropanoic acid **(F)**.

Strain F25 was inoculated into MSM medium spiked with DSF, and multiple time points were set for sampling, in order to investigate the pathway of DSF degradation by strain F25. Five metabolic products were identified during the biodegradation of DSF. **Figure S2** shows the results of the GC-MS analysis. A distinct peak was detected at a retention time (RT) of 17.459 min with a characteristic mass fragment [M+] at *m*/*z* = 99.0 and a major fragment ion at *m*/*z* = 43.1; this important compound was identified as DSF (**Figure 4A**). With time, the peaks of DSF decreased and a new compound appeared, at a RT of 15.003 min with *m*/*z* = 73.0 as the base peak, which was characterized as n-decanoic acid, based on the elution time and how well the molecular ion matched the corresponding authentic compound in the NIST database (**Figure 4B**). In addition to this, we detected several degradation products of DSF (**Figures 4C–F**). It is worth noting that these metabolites were transiently present. Finally, DSF was completely decomposed into carbon dioxide and water.

The metabolic pathway of DSF in strain F25 was proposed based on the intermediates formed during degradation and the chemical structure of DSF, as follows. First, the degradation of DSF begins with the oxidation of branched carbon atoms to form fatty acids with one less carbon atom. Then, the *cis-*double bond of the formed intermediate is converted to a *trans-*double bond by isomerase. Through β-oxidation of the fatty acid, the intermediate product forms a *trans-*2-decenoic acid with two fewer carbon atoms. The unsaturated fatty acids then undergo hydrogenation to form saturated fatty acids. Finally, these compounds disappeared with the complete degradation of DSF by strain F25.

### Antagonistic effect of strain F25 on XC1

Antagonism experiments were performed on strains F25 and XC1, in order to verify the presence of antagonism between them. LB agar plates containing pathogenic bacteria were prepared, and bacterial suspensions of strain F25 were injected into the plates. The results demonstrated that, when strain F25 was grown on the same plate as XC1, the plate showed no obvious transparent circle, indicating no zone of inhibition. The other two controls also showed no zone of inhibition. The antagonism experiment indicated that strain F25 has no antagonistic effect on *Xcc*.

### Study on the effectiveness of strain F25 in the biological control of black rot

**Figure 5:**
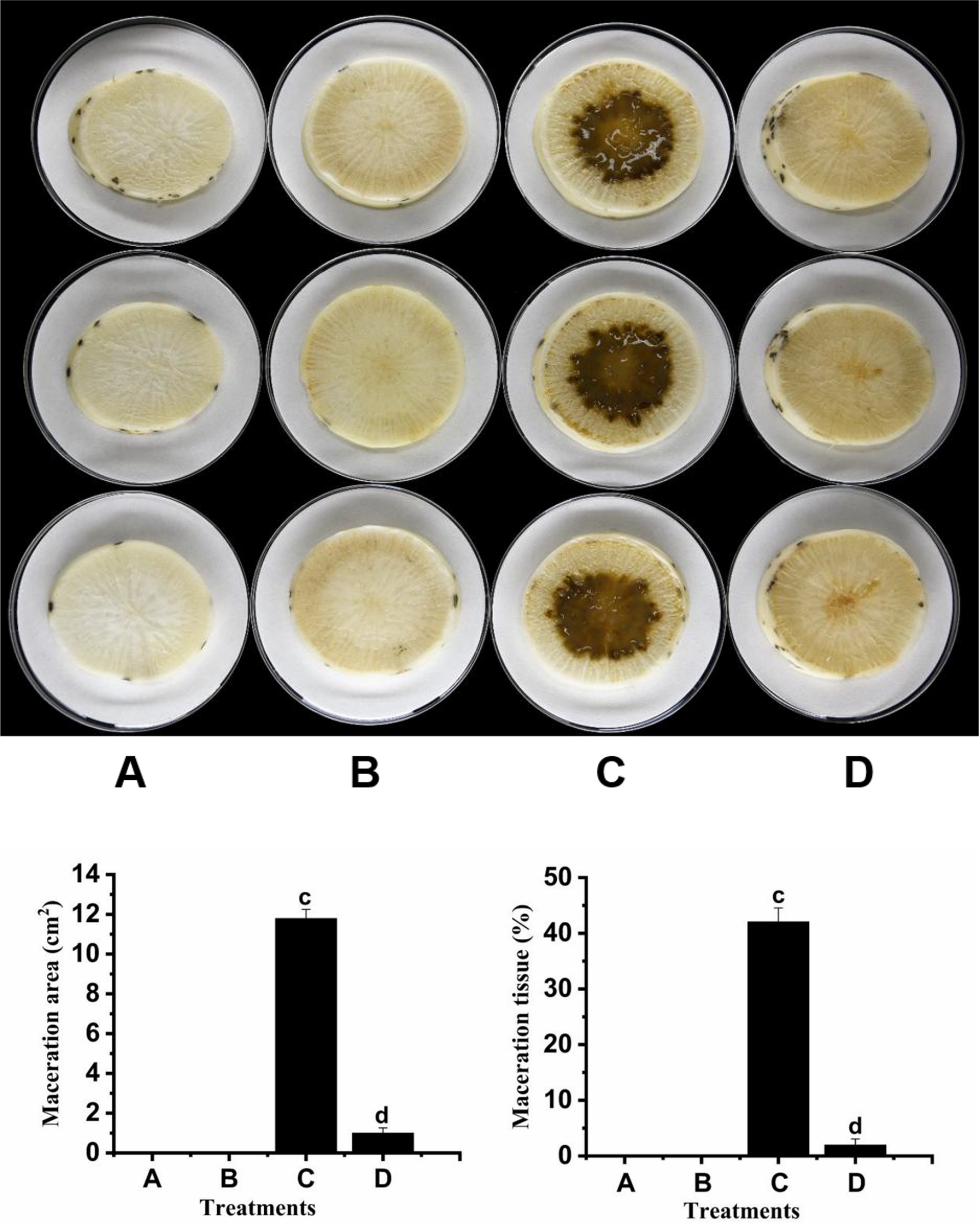
Biocontrol test of *Burkholderia* sp. F25 against black rot disease on radish slices under laboratory conditions. **(A)** shows radish root slices inoculated only with distilled water; **(B)** shows radish root slices inoculated only with the suspensions of DSF-degrading strain F25; **(C)** shows radish root slices inoculated only with the suspensions of black rot pathogen *Xcc*; **(D)** shows radish root slices co-inoculated with the suspensions of *Xcc* mixed with F25. Experimental data were analyzed by one-way analysis of variance (ANOVA), and means were compared by Bonferroni’s multiple comparison test using the GraphPad Prism software (Version 6.0). Experiments were arranged as a completely randomized design, and *P* < 0.05 was considered to indicate statistical significance. Values with different small letters are significantly different according to Duncan test at P < 0.05 level.

We tested the biocontrol ability of strain F25 against *Xcc* on radish slices, where the severity of the disease was reflected by the size of the maceration zone. The results are shown in **Figure 5**. The severity of the disease was high in radish slices treated with XC1 alone (**Figure 5C**). The dipping zone was significantly smaller in radish slices treated with the F25 and XC1 mixture (**Figure 5D**), while treatment of radish slices with strain F25 alone indicated that the strain was not harmful to the radishes (**Figure 5B**). Treatment of radish slices with only distilled water did not show any disease (**Figure 5A**). These results indicate that strain F25 exhibited good biological control potential against *Xcc* and, so, can be used as a biological agent to prevent infection by bacterial plant diseases.

### Biocontrol efficiency of F25 crude enzyme

**Figure 6:**
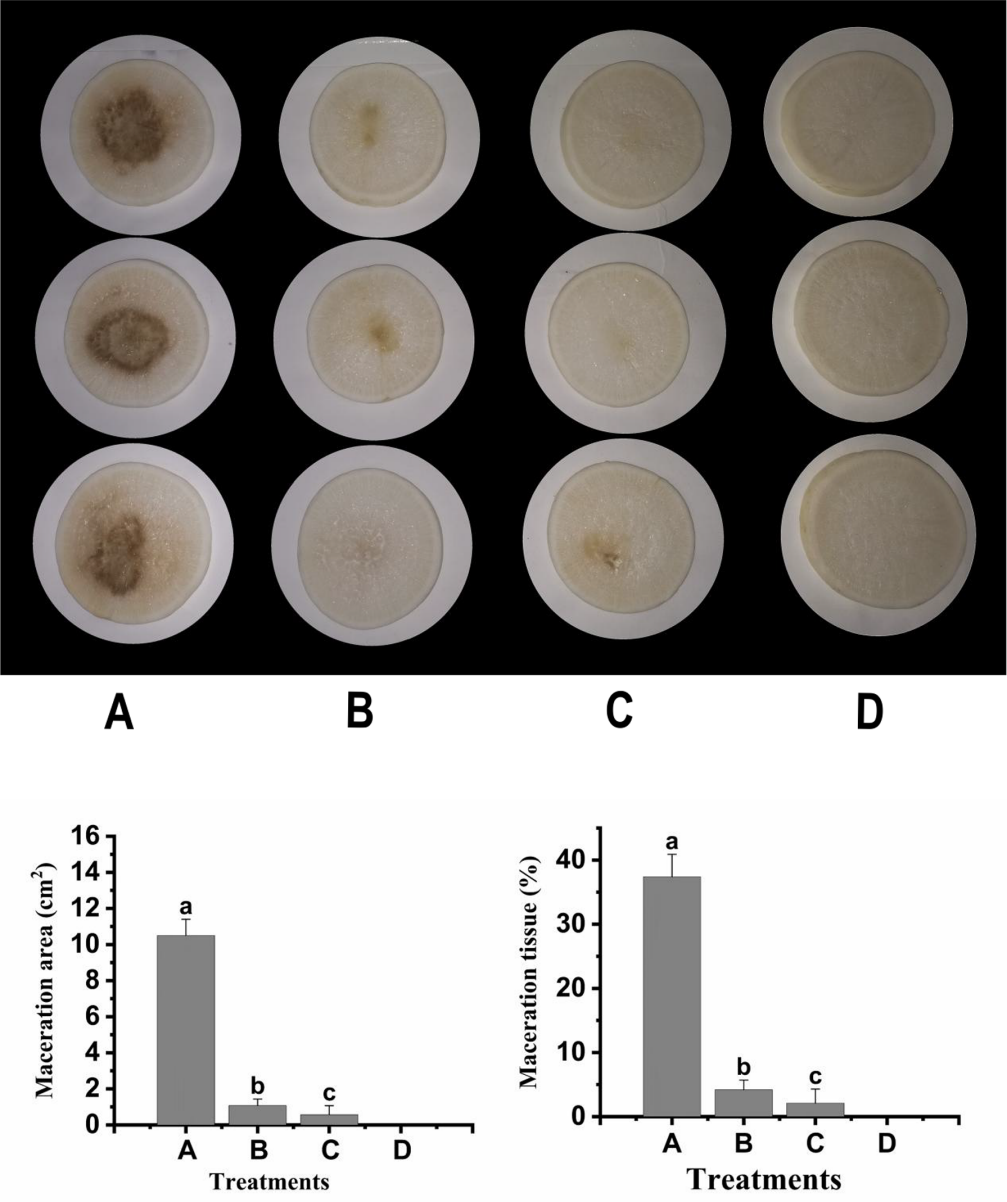
Preliminary biocontrol test of crude enzymes of *Burkholderia* sp. F25 against *Xanthomonas campestris* pv. *campestris* XC1: **(A)** Panel A, XC1 alone on plant slices; Panel **B**, XC1 + Intracellular enzyme; Panel **C**, XC1 + Extracellular enzyme; Panel **D**, Sterile water. **(B)** Maceration area (1) and maceration tissue (2) in each treatment. Experimental data were analyzed by one-way analysis of variance (ANOVA), and means were compared by Bonferroni’s multiple comparison test in the GraphPad Prism software (Version 6.0). Experiments were arranged as a completely randomized design, and *P* < 0.05 was considered statistically significant. Values with different small letters are significantly different according to Duncan test at P < 0.05 level.

To verify the biocontrol effect of strain F25 crude enzyme on *Xcc*, the pathogenic bacteria XC1, intracellular enzyme, and extracellular enzyme were inoculated onto radish slices under ex vivo conditions and incubated for 48 h. The experimental results are shown in **Figure 6**, where a slight decay was observed in the crude enzyme-treated group; however, the area of the macerated zone was significantly lower, compared to the XC1-treated group. These results indicate that the crude enzyme of strain F25 also has a good biocontrol effect and has great potential to be developed for the treatment of plant diseases caused by *Xcc*.

### F25 has acylase activity

To verify whether strain F25 presents acylase activity, intracellular and extracellular enzymes of strain F25 were extracted and tested using an analytical kit. From the results, we observed that the control group was colorless, while the experimental group with the addition of the intracellular enzyme exhibited a distinct yellow color (**Figure S3**), indicating that strain F25 possesses acylase activity.

## Discussion

QS acts as a communication mechanism for microorganisms, regulating cell growth, secretion of pathogen virulence factors, and biofilm formation (Miller and Bassler, 2001; Deng et al., 2013a; Xu et al., 2021). All of these regulatory processes help microorganisms to cause disease and, thus, have a significant impact on agricultural production, the environment, and human health (Deng et al., 2013b; Whiteley et al., 2018). The QQ pathway can be used to inhibit the expression of pathogenic genes in the QS process by degrading the signaling molecules produced during the QS process by QQ bacteria or QQ enzymes (Fan et al., 2020), such that the signaling molecules do not reach a certain threshold value, thus blocking intercellular communication between the pathogenic bacteria (Boon et al., 2008). To date, studies on QQ bacteria have mainly focused on AHL-mediated QS family systems, and only a few studies have reported on the role of DSF-degrading bacteria in the control of black rot caused by *Xcc* in wild rape (Huedo et al., 2018; Samal and Chatterjee, 2019). A large number of microorganisms that can degrade signaling molecules exist in nature, which has the advantages of being diverse, abundant, and easy to cultivate (Zhang et al., 2021). Therefore, screening QQ bacteria from nature for biodegradation applications has obvious advantages.

In this study, we isolated a new QQ strain from an agricultural field, where morphological characteristics and 16S rDNA phylogenetic analysis of the strain identified it as a member of *Burkholderia*. Strain F25 was isolated from the soil of a perennial cultivated sweet potato field, and was well-adapted to the environment. QQ strain F25 can degrade DSF rapidly and efficiently and possesses acylase activity, as well as having a metabolic pathway by which DSF can be completely degraded and metabolized. Our experimental results showed that strain F25 can degrade DSF in a short period of time and can significantly reduce the function of DSF-dependent diseases to achieve a biological control effect. Previous studies have demonstrated that *Burkholderia* strains have strong metabolic capability and environmental versatility as well as excellent ability to manage bacterial and fungal pathogens infecting crop plants (Mannaa et al., 2018; Ye et al., 2020a; Pal et al., 2022). Furthermore, a new degradation pathway of DSF in the presence of microorganisms was proposed, based on the metabolites of isolate F25 detected by GC-MS and the chemical structure of DSF. First, to promote *β*-oxidation, DSF is converted from *cis* to *trans* double bonds, as catalyzed by enzymes, where the methyl groups on fatty acids are oxidized. Immediately afterward, *β*-oxidation continues to form intermediate products with two fewer carbon atoms. The unsaturated fatty acids are then hydrogenated to form saturated fatty acids. DSF is eventually degraded to carbon dioxide and water by microbial metabolism, without any long-term accumulation of intermediates. In addition, strain F25 presented significant resistance to gentamicin, neomycin sulfate, carbenicillin, ampicillin, and streptomycin.

Inoculation experiments were conducted to evaluate the biocontrol effect of strain F25. The results showed that strain F25 did not cause the development of other diseases and also had a good biocontrol effect against black rot caused by *Xcc* in plants. The above experiments demonstrated that strain F25 and its crude enzyme can be applied as effective biocontrol agents for controlling plant diseases caused by DSF-dependent bacterial pathogens, and the great potential of strain F25 for the control of DSF-mediated pathogenic bacterial damage was demonstrated. This provides a new avenue for the development of a treatment strategy that replaces chemical control with biological control, allowing for the blockage of QS without causing selection pressure.

## Conclusions

In summary, we identified a novel quorum quencher, *Burkholderia* sp. F25, which can be used as a new QQ strain and has excellent DSF degradation capacity. Strain F25 possesses acylase activity and a pathway for complete DSF degradation and metabolism. In addition, strain F25 and its crude enzyme were able to significantly attenuate black rot in plants, and thus could be used as potential biocontrol agents against plant diseases caused by DSF-dependent bacterial pathogens. This work provided a biochemical basis for the efficient DSF-degrading activity of strain F25, and offers new perspectives for further studies on the suppression mechanisms of plant pathogens. In the future, in-depth studies on the gene clusters of strain F25 related to DSF degradation are required, in order to elucidate the genetic mechanisms of strain F25 and to improve its stability in practical applications.

## Data availability statement

The original contributions presented in the study are included in the article/**Supplementary Materials**. Further inquiries can be directed to the corresponding authors.

## Author contributions

L-HZ, SC, HW, and LL conceived the presented idea. HY, W-JC, and JH contributed to the writing and prepared the figures. KB, ZZ, XZ, SL, SC, HW, and LL participated in revising the manuscript. All authors contributed to the article and approved the submitted version.

## Funding

This work was funded by the National Natural Science Foundation of China (31900076; 32200025), the Key Realm R&D Program of Guangdong Province, China (2020B0202090001), Guangdong Province Key Laboratory of Microbial Signals and Disease Control Open Grant, China (MSDC2017-13), the Climbing Project of Guangdong Province, China (pdjh2022a0073), and National College Students' Innovation and Entrepreneurship Training Program, China (202110564076).

## Conflict of interest

The authors declare that the research was conducted in the absence of any commercial or financial relationships that could be construed as a potential conflict of interest.

The handling editor PB declared a past co-authorship with the authors. The funders had no role in the design of the study, in the collection, analyses, or interpretation of data, in the writing of the manuscript, or in the decision to publish the results.

## Publisher’s note

All claims expressed in this article are solely those of the authors and do not necessarily represent those of their affiliated organizations, or those of the publisher, the editors and the reviewers. Any product that may be evaluated in this article, or claim that may be made by its manufacturer, is not guaranteed or endorsed by the publisher.
