# Supplementary material for: A novel bacterial strain Burkholderia sp. F25 capable of degrading diffusible signal factor signal shows strong biocontrol potential
Source: Front Plant Sci. 2022 Nov 24;13:1071693. doi: 10.3389/fpls.2022.1071693 (PMC9730286; doi:10.3389/fpls.2022.1071693)
Supplement: Supplementary file 1 [file DataSheet_1.doc]

**A novel bacterial strain *Burkholderia* sp. F25 capable of degrading diffusible signal factor signal shows strong biocontrol potential**

Hongxiao Yu^1#^, Wen-Juan Chen^1,2#^, Kalpana Bhatt^3^, Zhe Zhou^1,2^, Xixian Zhu^1,2^, Siqi Liu^1^, Jiehua He^1^, Lian-Hui Zhang^1,2^, Shaohua Chen^1,2*^, Huishan Wang^1,2*^, Lisheng Liao^1,2*^

^1^Department of Plant Pathology, College of Plant Protection, South China Agricultural University, Guangzhou 510642, China

^2^Guangdong Province Key Laboratory of Microbial Signals and Disease Control, Integrative Microbiology Research Centre, South China Agricultural University, Guangzhou 510642, China

^3^Department of Botany and Microbiology, Gurukula Kangri University, Haridwar 249404, Uttarakhand, [India](mailto:India.Email.kalpana.kanubhatt@gmail.comPleasefindtheattachedfiles.Regards)

# Both authors contributed equally to this work.

*Correspondence: shchen@scau.edu.cn (S.C.); [499711038@qq.com (H.W.)](mailto:junxiawang@scau.edu.cn;); lishengliao@scau.edu.cn (L.L.). Tel: +86-20-8528 8229.


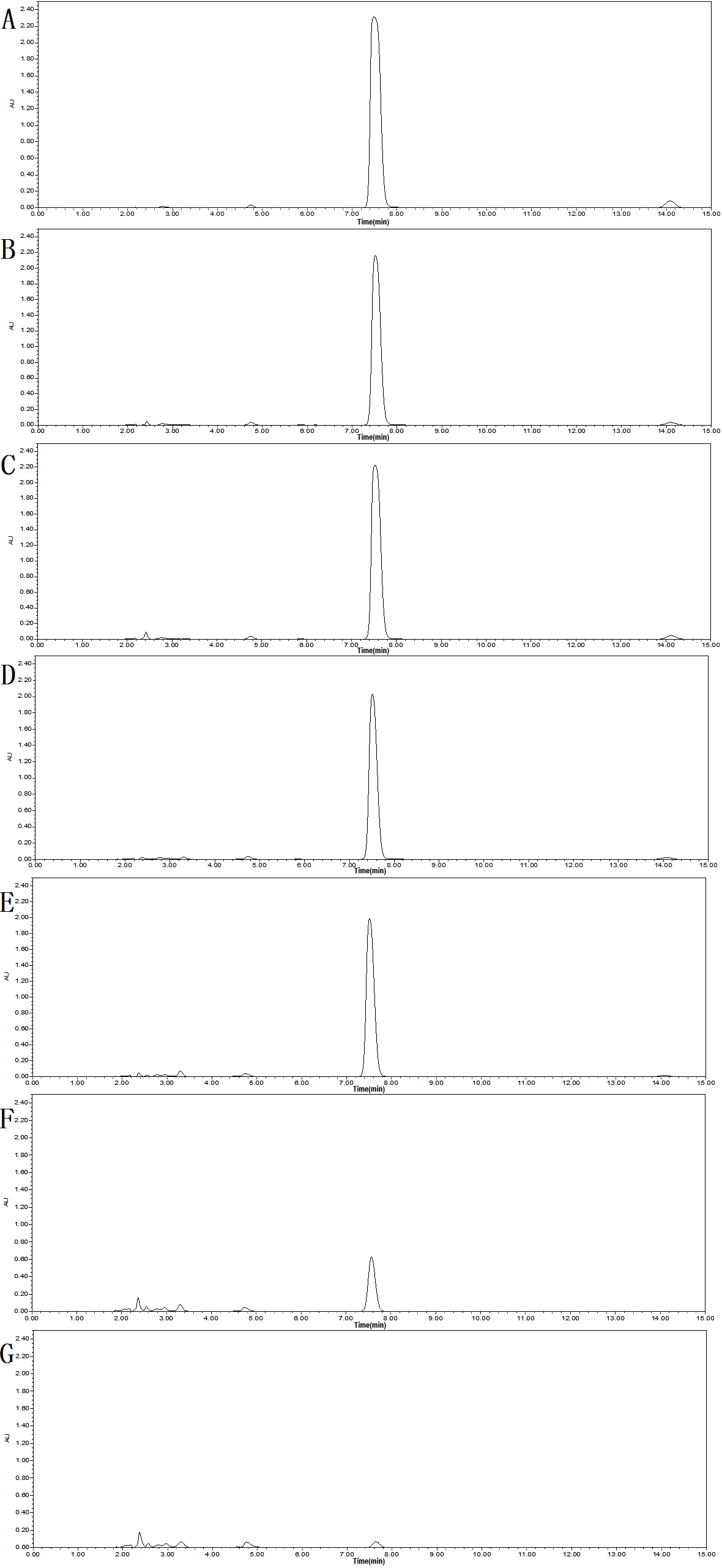


**Figure S1.** The remaining amount of DSF at different time intervals was determined by high-performance liquid chromatography (HPLC): (A) Mineral salt medium (MSM) with DSF alone as a control. DSF degradation by the strain *Burkholderia* sp. F25 at 12 (B), 24 (C), 36 (D), 48 (E), 60 (F), and 72 h (G), respectively.


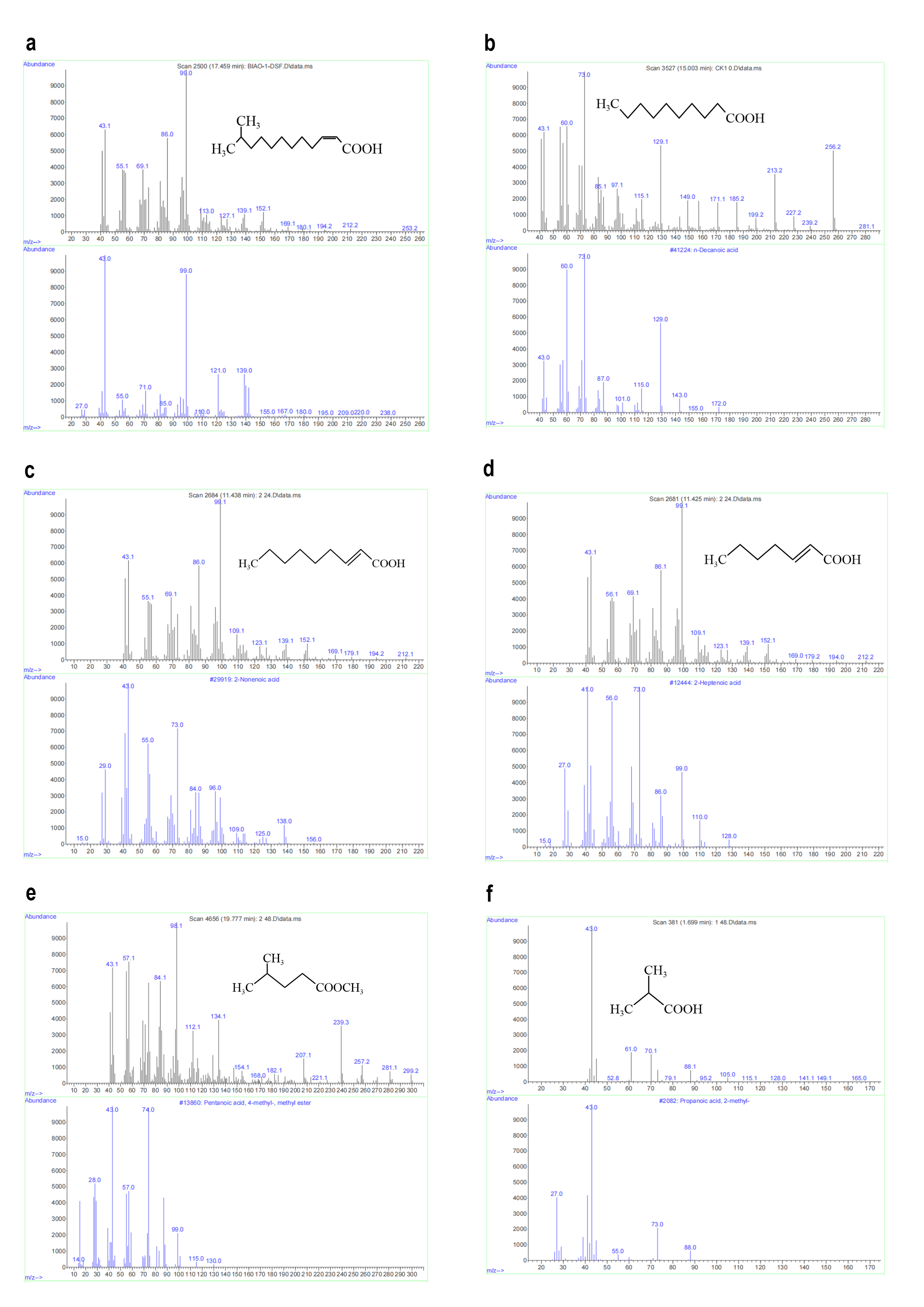
**Figure S2.** Elution times and molecular ions of DSF and its degradation products compared to the corresponding authentic standard compounds in the NIST database: DSF (a); *N-*decanoic acid (b); *trans-*2-nonenoic acid (c); *trans-*2-heptenoic acid (d); methyl 4-methylpentanoate (e); and 2-methylpropanoic acid (f).


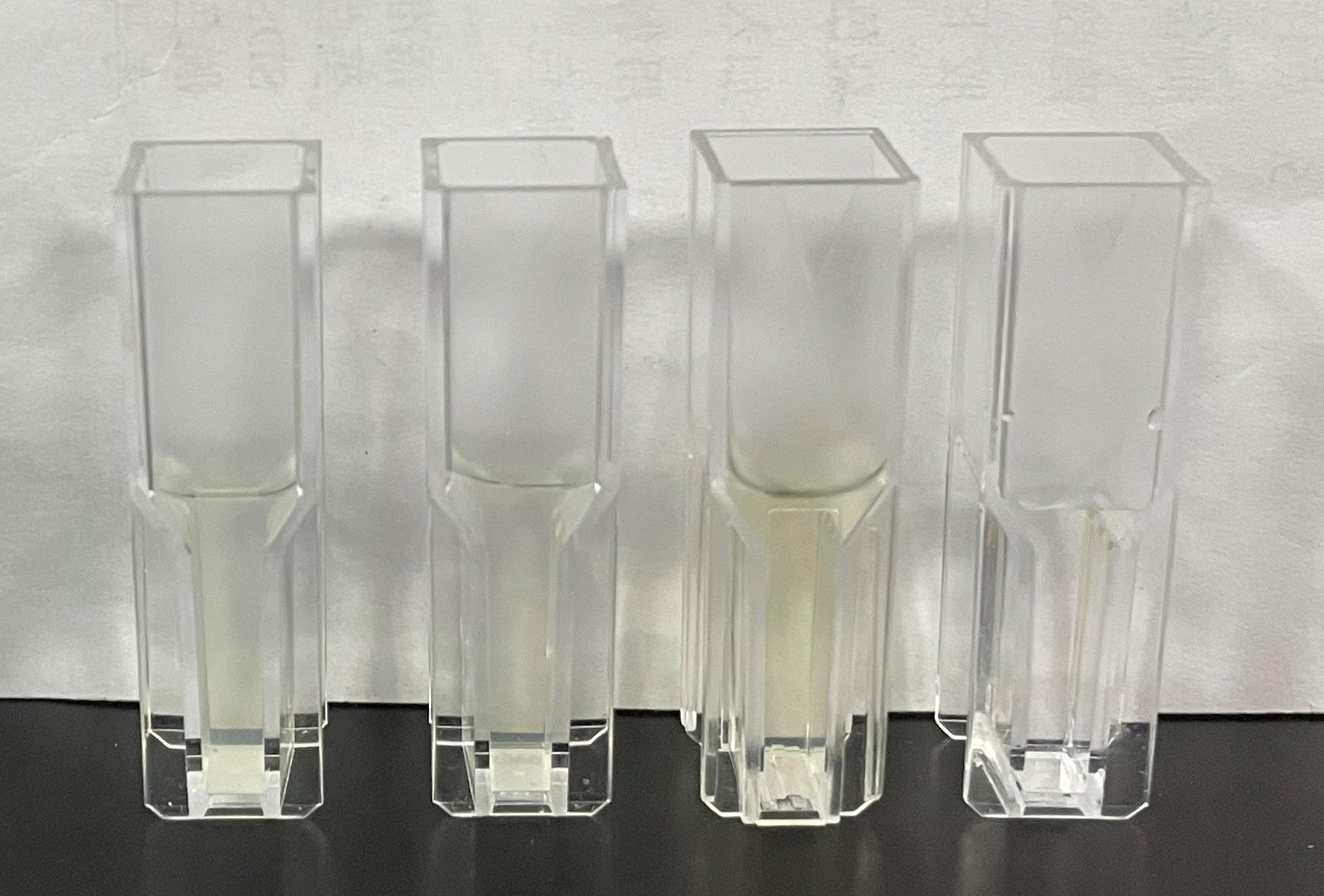


A B C D

**Figure S3.** Acylase activity test: A, blank control (distilled water); B, negative control (blank reagent without enzyme); C, intracellular enzymes; D, extracellular enzymes. Acylase can catalyze the transfer of the acetyl group of acetyl-CoA to butanol and, at the same time, reduce 5,5'-dithiobis-(2-nitrobenzoic acid) (DTNB) to generate 2-nitro-5-thiobenzoic acid (TNB). The TNB compound appears yellow, with an absorption peak at 412 nm.
